# Homozygous microdeletion of exon 5 in *ZNF277* in a girl with specific language impairment

**DOI:** 10.1038/ejhg.2014.4

**Published:** 2014-02-12

**Authors:** Fabiola Ceroni, Nuala H Simpson, Clyde Francks, Gillian Baird, Gina Conti-Ramsden, Ann Clark, Patrick F Bolton, Elizabeth R Hennessy, Peter Donnelly, David R Bentley, Hilary Martin, IMGSAC,, SLI Consortium,, WGS500 Consortium,, Jeremy Parr, Alistair T Pagnamenta, Elena Maestrini, Elena Bacchelli, Simon E Fisher, Dianne F Newbury

**Affiliations:** 1 Dipartimento di Farmacia e Biotecnologie, University of Bologna, Bologna, Italy; 2 Wellcome Trust Centre for Human Genetics, University of Oxford, Oxford, UK; 3 Max Planck Institute for Psycholinguistics, Nijmegen, Netherlands; 4 Donders Institute for Brain, Cognition & Behaviour, Nijmegen, Netherlands; 5 Guy's & St Thomas NHS Foundation Trust, Newcomen Children's Neurosciences Centre, St Thomas' Hospital, London, UK; 6 School of Psychological Sciences, The University of Manchester, Manchester, UK; 7 Speech and Hearing Sciences, Queen Margaret University, Edinburgh, UK; 8 Departments of Child & Adolescent Psychiatry & Social Genetic & Developmental Psychiatry Centre, Institute of Psychiatry, Kings College London, London, UK; 9 University Child Health and DMDE, University of Aberdeen, Aberdeen, UK; 10 Illumina Cambridge Ltd., Chesterford Research Park, Little Chesterford, Essex, UK; 11 Institute of Neuroscience and Health and Society, Newcastle University, Newcastle, UK; 12 NIHR Biomedical Research Centre, Oxford and Wellcome Trust Centre for Human Genetics, University of Oxford, Oxford, UK

**Keywords:** *ZNF277*, SLI, language

## Abstract

Specific language impairment (SLI), an unexpected failure to develop appropriate language skills despite adequate non-verbal intelligence, is a heterogeneous multifactorial disorder with a complex genetic basis. We identified a homozygous microdeletion of 21,379 bp in the *ZNF277* gene (NM_021994.2), encompassing exon 5, in an individual with severe receptive and expressive language impairment. The microdeletion was not found in the proband's affected sister or her brother who had mild language impairment. However, it was inherited from both parents, each of whom carries a heterozygous microdeletion and has a history of language problems. The microdeletion falls within the *AUTS1* locus, a region linked to autistic spectrum disorders (ASDs). Moreover, *ZNF277* is adjacent to the *DOCK4* and *IMMP2L* genes, which have been implicated in ASD. We screened for the presence of *ZNF277* microdeletions in cohorts of children with SLI or ASD and panels of control subjects. *ZNF277* microdeletions were at an increased allelic frequency in SLI probands (1.1%) compared with both ASD family members (0.3%) and independent controls (0.4%). We performed quantitative RT-PCR analyses of the expression of *IMMP2L*, *DOCK4* and *ZNF277* in individuals carrying either an *IMMP2L*_*DOCK4* microdeletion or a *ZNF277* microdeletion. Although *ZNF277* microdeletions reduce the expression of *ZNF277*, they do not alter the levels of *DOCK4* or *IMMP2L* transcripts. Conversely, *IMMP2L*_*DOCK4* microdeletions do not affect the expression levels of *ZNF277*. We postulate that *ZNF277* microdeletions may contribute to the risk of language impairments in a manner that is independent of the autism risk loci previously described in this region.

## Introduction

Specific language impairment (SLI) is a common neurodevelopmental disorder diagnosed in children with language abilities below age expectations, given adequate educational opportunities and in the absence of other medical conditions, such as hearing deficits or intellectual disability. SLI affects 3–7% of preschool English-speaking children.^1^ Twin and family studies support the role of a strong genetic background in SLI,^2^ but a complex inheritance pattern suggests that several loci and environmental factors contribute to the overall risk.

Although a diagnosis of SLI excludes the presence of other clinical conditions that affect language, SLI presents co-morbidity with other neurodevelopmental disorders, such as autism spectrum disorder (ASD). Although ASD is characterized by an impaired communication, language skills can be highly heterogeneous and phenotypic overlaps with SLI in the language domain have been debated. Some studies report that a minority of adolescents with a history of SLI show behavioral traits reminiscent of autism.^3, 4, 5^ Similarly, some find that a subset of children with ASD exhibit language profiles resembling SLI.^6, 7^ However, others report qualitative differences that can distinguish the performance of individuals with SLI or ASD in language tests.^8, 9, 10^

Recent studies investigated whether the phenotypic similarities of the language deficits may reflect potentially shared causes. An example of functional link between SLI and ASD, in addition to other neurodevelopmental disorders, is given by *CNTNAP2*: variants in and disruptions of this gene are reported to be associated with language endophenotypes in both SLI and ASD,^11^ suggesting that *CNTNAP2* might harbour susceptibility risk factors that could impair language skills in distinct language-related disorders.

Submicroscopic copy number variations (CNVs) have been shown to be an important source of susceptibility to psychiatric disorders,^12^ such as ASD, schizophrenia and bipolar disorder. Some CNVs are recurrently observed across multiple neuropsychiatric conditions,^13^ sometimes with variable penetrance of the genes implicated, suggesting a pleiotropic effect. These findings support the hypothesis that these CNVs can represent shared risk variants across different types of neurodevelopmental diseases and might highlight common genes and/or pathways.

Here, we describe a novel CNV case involving homozygous loss of material from the *ZNF277* gene, found in a child with SLI. This CNV falls in the *AUTS1* region of linkage to ASD (7q21-q32, OMIM#209850).^14, 15, 16, 17, 18^ A fine mapping study of *AUTS1* previously reported association of ASD with SNPs in two genes that are proximal to *ZNF277*, *DOCK4* (dedicator of cytokinesis 4, OMIM#607679) and *IMMP2L* (IMP2 inner mitochondrial membrane protease-like, OMIM#605977).^19^ An additional investigation^20^ described a rare microdeletion involving the *DOCK4* and *IMMP2L* genes that co-segregated with the presence of dyslexia in an extended family.

On the basis of prior reports of phenotypic and genetic overlaps between SLI and ASD, and given the position of this homozygous microdeletion, we hypothesized that *ZNF277* may represent a candidate gene for both disorders. Therefore, we went on to investigate the frequency of *ZNF277* microdeletions in cohorts of individuals with SLI or ASD. Finally, we used quantitative PCR (qPCR) techniques to further examine the effects of *ZNF277* microdeletions on expression levels of genes in the *AUTS1* region.

## Materials and methods

### Discovery pedigree

A genome-wide CNV screen of 512 individuals from families with SLI was performed to investigate CNV burden in individuals with SLI. This study utilized genome-wide SNP data from the Illumina Human OmniExpress (v12.1) beadchip and identified CNVs within the Nexus,^21^ PennCNV^22^ and QuantiSNP^23^ algorithms. A manuscript describing this larger data set is in preparation. In the current manuscript, we describe an incidental finding from these data: the identification of a homozygous microdeletion involving exon 5 of *ZNF277* (NM_021994.2) (chr7:g111955948_111960100del, hg19). As this microdeletion was in a homozygous form, we predicted that it might be particularly deleterious to the individual and thus selected it for further study, as described below.

The microdeletion was predicted to be present in a single child of a non-consanguineous Caucasian family. The child had a clinical diagnosis of SLI. She did not develop language skills until the age of 4–5 years. She appeared sociable but was dependent on being shown what to do with toys. Her thinking was slightly rigid but no other obvious autistic behaviors were reported or observed. Her non-verbal intelligence was below average (performance IQ=75). She attended a special unit for speech and language impaired children.

The proband had two siblings, an older brother and a younger sister. All three children presented with a similar pattern of speech and language impairment, which primarily affected the expressive domain. They all presented with delayed word and phrase speech, unintelligible speech with poor articulation and impaired word retrieval. However, the three children differed in terms of the severity of their impairment and their non-verbal attainment. The younger sister had a slightly higher non-verbal IQ than the proband (PIQ=94) and also had a diagnosis of SLI, although she appeared less severely affected than the proband. The brother had a particularly high non-verbal IQ (PIQ=127) and although he was reported to have had an early speech and language delay, he did not have a diagnosis of SLI. He did attend a special educational unit and at 10 years of age he had a significant verbal performance discrepancy and impaired sentence recall. Both parents reported a family history of speech or language problems.

### Validation in the discovery pedigree

qPCR using iQ SYBR-green Supermix (Bio-Rad, Hercules, CA, USA) and four primer pairs across the predicted CNV and the surrounding region was used to validate the presence of the microdeletion and to examine co-segregation in the discovery pedigree. Primer sequences are available on request. All the members of the family were analyzed by qPCR, except for the sister, for whom insufficient DNA was available. The sister was instead tested using a PCR assay with primers spanning the *ZNF277* microdeletion breakpoints, as described for the screening of the larger cohort below. For each sample, qPCRs were set up in triplicate and compared against a control gene (*ZNF423*) and a control subject. The number of copies of each amplified fragment was calculated using the 2^ΔΔCt^ method.^24^

The boundaries of the *ZNF277* microdeletion were determined by Sanger sequencing using primers flanking the predicted deletion boundaries.

### Screening of larger cohorts

Primers spanning the *ZNF277* microdeletion breakpoints, which generated a shortened product in the presence of the microdeletion, were used for a PCR-based screening of three separate cohorts: a cohort of families containing individuals with SLI, a cohort of families containing individuals with ASD and a control cohort. A second primer pair that amplifies exon 5 of *ZNF277*, giving a product only when the allele is not deleted, was subsequently used to test whether the identified microdeletions were in the heterozygous or homozygous form.

The SLI screening cohort consisted of DNA from 1234 individuals from 322 families (545 parents, 318 SLI probands and 371 siblings). This cohort included the 512 individuals who comprised the CNV study but included many additional SLI subjects (144 additional probands) and their family members (550 individuals). All families formed a part of the SLI Consortium cohort, which has previously been described in detail.^25, 26, 27, 28^ In short, these British nuclear families were ascertained on the basis of at least one child who had expressive or receptive language skills ≥1.5 standard deviations (s.d.) below the normative mean for their chronological age and WISC Perceptual Organisation Index (a composite score of the non-verbal subtests Picture Completion, Picture Arrangement, Block Design and Object Assembly) of >77.5 (1.5 s.d. below that expected for their age).

The ASD cohort consisted of DNA from 1021 individuals from 252 families (454 parents, 412 affected children and 155 siblings) and formed part of the International Molecular Genetic Study of Autism Consortium (IMGSAC) cohort, which has previously been described.^16^ The families were collected from different countries (UK, Netherlands, France, USA, Germany, Denmark and Greece) and were predominantly Caucasian (92.5%). In short, this cohort consists of multiplex ASD families in which at least one child meets a clinical diagnosis of ASD under the Autism Diagnostic Interview and/or Autism Diagnostic Observation Schedule (ADOS) or ADOS generic.

The control cohort consisted of DNA from 224 non-related UK Caucasian blood donors from the ECACC Human Random Control (HRC) panel (http://www.hpacultures.org.uk/products/dna/hrcdna/hrcdna.jsp). In addition, we had access to sequence data from 130 unrelated Caucasian samples through an in-house project at the Wellcome Trust Centre for Human Genetics – the 500 Whole-Genome Sequences Project (WGS500 Consortium^29^).

We used the two-tailed Fisher's exact test (1 degree of freedom) to test whether the allelic frequency of *ZNF277* microdeletions was significantly different between SLI probands and control individuals.

### Gene expression evaluation

RNA samples were not available for the discovery individuals. We therefore chose to examine expression levels of *ZNF277*, *DOCK4* and *IMMP2L* by qPCR in cDNA derived from lymphoblastoid cell lines from 10 individuals belonging to four ASD families, 5 of whom carried a heterozygous *ZNF277* microdeletion, and the expression level of *ZNF277* in cDNA derived from blood from two individuals from a single Dutch multiplex ASD family previously described by Pagnamenta *et al.*^20^ with an *IMMP2L*_*DOCK4* microdeletion (chr7:g.110876742_111470446del, hg19). The housekeeping gene *GUSB* (NM_000181.3) was used as a normalizer and expression levels were normalized against a control individual. The 2^ΔΔCt^ method was applied to estimate the difference in the expression of the three genes between the samples.^24^ Statistical significance was calculated with the Student *T*-test, assuming unequal variance between the two independent sample groups for the expression analysis.

## Results

### Identification and validation of a homozygous microdeletion of exon 5 in *ZNF277*

During a CNV analysis of SNP array data, a female patient with a diagnosis of SLI was found to carry a novel homozygous microdeletion within the gene *ZNF277*. The microdeletion removes exon 5 of the *ZNF277* gene, causing a frame-shift mutation and introducing a premature stop codon in exon 7 (NM_021994.2). Given the likelihood of non-sense-mediated mRNA decay, this homozygous microdeletion would thus be predicted to result in a complete lack of functional protein in the affected individual.^30^

**Figure 1:**
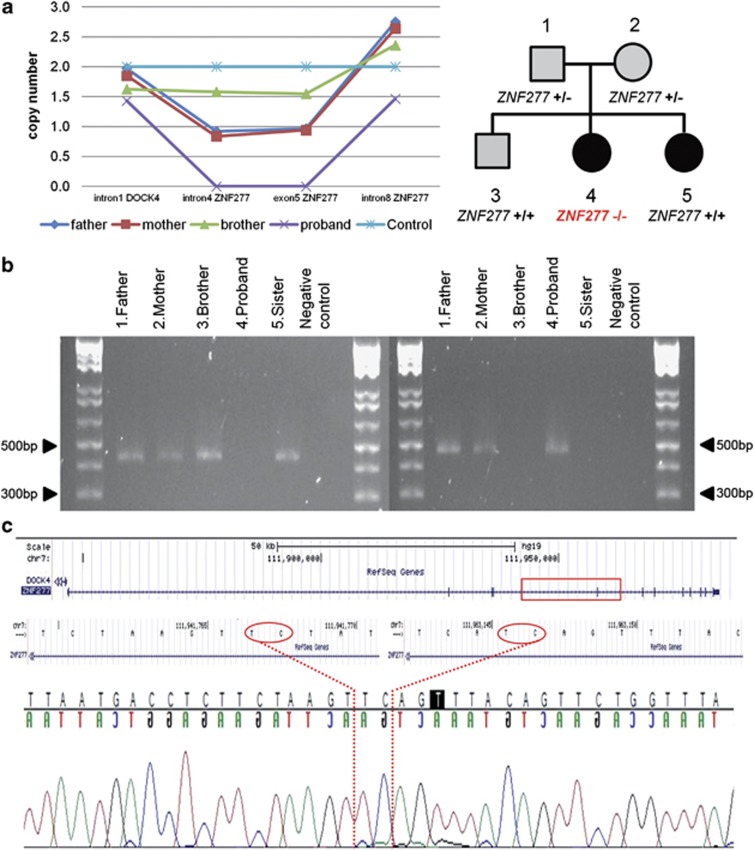
Molecular characterization of the *ZNF277* microdeletion in the discovery pedigree. (**a**) On the left, the results from the qPCR, on the right the discovery pedigree, where black indicates diagnosis of SLI and grey indicates language problems. (**b**) On the right, the results of PCR across the microdeletion breakpoints: only the allele with the microdeletion can be amplified and visualized as a band of 466 bp in the parents and in the proband. On the left, amplification of exon 5 of *ZNF277* indicates the presence of at least one allele without the microdeletion. In both gels, 1 kb Plus DNA ladder was loaded at the extremities. (**c**) Sequence electropherograms from the PCR products spanning the microdeletion in *ZNF277*. The rectangle indicates the genomic position of the microdeletion in *ZNF277*. The 2 bp (TC), common to both ends, are delimited by dotted lines and circled in the reference sequence. bp, base pair, PCR, polymerase chain reaction, qPCR, qPCR.

qPCR of available family members in this pedigree demonstrated that one copy of the microdeletion was transmitted to the proband from each parent, who were both heterozygotes and reported to have language problems in their childhood: the father had speech impairment and the mother had dyslexia. However, the microdeletion was not transmitted to the proband's brother, who presented with an early expressive speech and language impairment but did not have a diagnosis of SLI (Figure 1a). Insufficient DNA was available for the qPCR assay in the proband's sister.

The predicted microdeletion included only three SNP probes (rs11769219, rs4727766 and rs7802828) and had a minimum predicted size of 4153 bp. At the time of detection, there were no overlapping deletions described in the Database of Genomic Variants (DGV)^31, 32^ (January 2012). Subsequently, one larger, overlapping heterozygous loss, was described in a HapMap female control sample (NA12156) (DGV, February 2013). A similar size microdeletion was observed in an in-house sequencing database at the Wellcome Trust Centre for Human Genetics in a heterozygous form (1/130 samples of the 500 Whole-Genome Sequences Project (WGS500 Consortium^29^)) allowing the accurate detection of the breakpoint boundaries which lie in two LINE elements, L2c and L1M4. Sanger sequencing validated the boundaries in the discovery individual (chr7:g.111941769_111963147, hg19: 21 379 bp) and confirmed the presence of the microdeletion in the parents and proband but not in either of the siblings (Figures 1b and c).

### Detection of the *ZNF277* heterozygous microdeletion in the SLIC and IMGSAC cohorts

**Figure 2:**
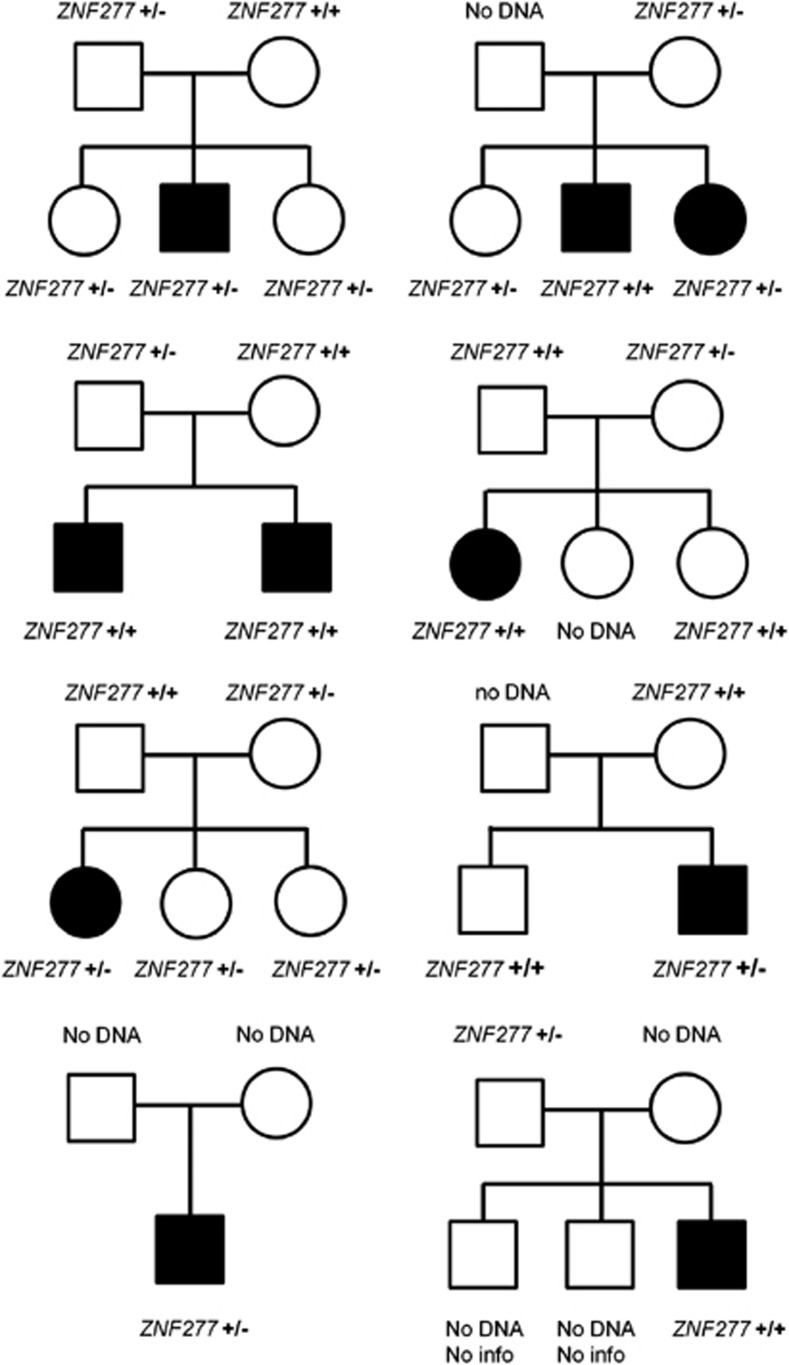
Pedigree of the SLIC families carrying the *ZNF277* microdeletion. Black filling indicates full diagnosis of SLI.

In addition to the discovery family, another 1229 individuals were screened giving a total cohort size of 322 families (1234 individuals) affected by SLI (545 parents, 318 probands and 371 siblings). The screening led to the identification of 16 additional individuals with the *ZNF277* microdeletion in a heterozygous form, 5 of whom were probands (allelic frequency 0.8%), 6 parents (allelic frequency 0.6%) and 5 siblings (allelic frequency 0.7%) (Figure 2), giving an allelic frequency of 0.8% in the entire cohort (20/2468 chromosomes). Across all SLI probands (ie independent cases including the discovery proband), the allelic frequency of microdeletions was therefore 1.1% (7/636 chromosomes).

In contrast, the microdeletion was observed in the heterozygous form in 1 of 130 unrelated samples in our in-house sequencing cohort (allelic frequency 0.4%) and 2 of 224 ECACC control individuals (allelic frequency 0.4%) giving a control population allelic frequency of 0.4% (3/708 chromosomes).

Screening of 252 ASD families (1021 individuals) identified heterozygous *ZNF277* microdeletions in 4 ASD families. Four mothers carried the microdeletion (allelic frequency 0.4%) and it was inherited by three affected children (allelic frequency 0.4%) giving a frequency of 0.3% (7/2042 chromosomes) across the entire cohort. All of the ASD families were ascertained as multiplex pedigrees and thus included more than one affected child. Unlike the SLI families, in many cases, there was no single designated proband within the family units. All three ASD cases who inherited the microdeletion had affected siblings who did not inherit the microdeletion rendering the derivation of an objective proband frequency problematic.

### The deletion of exon 5 in *ZNF277* causes a decrease in the mRNA expression

**Figure 3:**
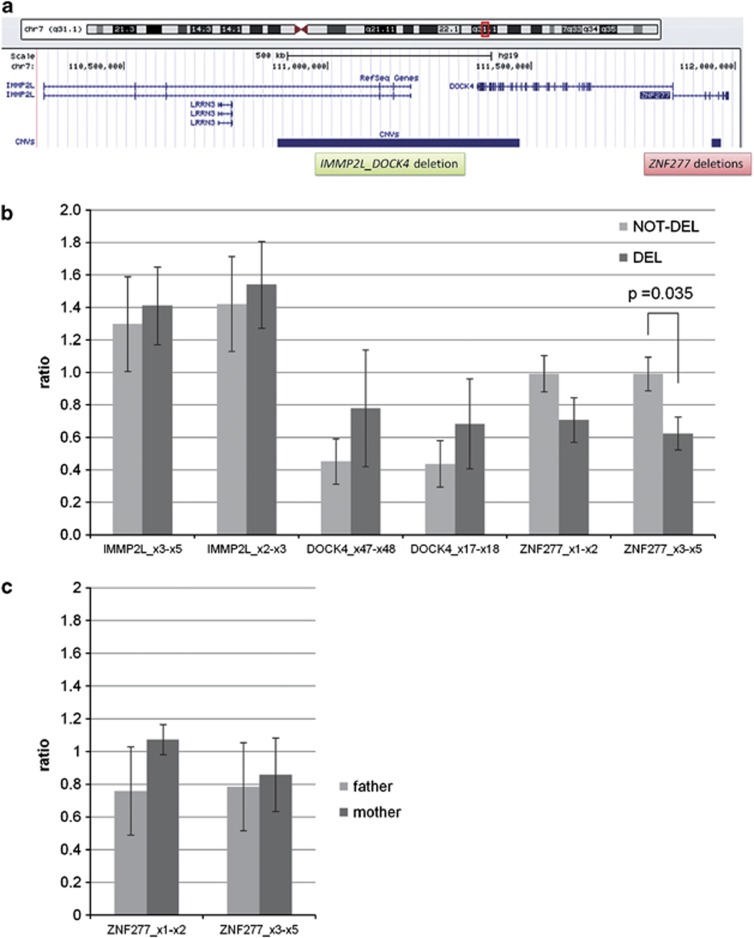
*IMMP2L*, *DOCK4* and *ZNF277* transcription levels in individuals with *ZNF277* microdeletion. *ZNF277* transcription levels in individuals with *IMMP2L_DOCK4* microdeletion. The graph shows the ratio of *IMMP2L*, *DOCK4* and *ZNF277* transcript levels, normalized using *GUSB* as a reference. (**a**) Schematic representation showing *ZNF277, DOCK4* and *IMMP2L* loci with respect to chromosome 7. The bars underneath show the chromosomal position of the two types of microdeletions that were analyzed. (**b**) the ratio has been calculated as an average of five samples for each group of individuals, belonging to four ASD families: ‘not-del' indicates the group of individuals with two wild-type copies of *ZNF277*, ‘del' the group of individuals with the heterozygous *ZNF277* microdeletion. Two fragments were tested for the *ZNF277* transcript: exons 1–2 (which lies outside of the microdeletion) and exons 3–5 (which encompasses the microdeletion). The expression pattern for the fragments was decreased in both cases when compared with individuals without the CNV, whereas in exon 5 it was significantly lower (*P*=0.035). Bars indicate the standard errors. (**c**) The ratio of *ZNF277* expression has been calculated for two individuals of a family with an *IMMP2L_DOCK4* microdeletion: the father, who has two normal copies of *IMMP2L* and *DOCK4*, and the mother, who carries the *IMMP2L_DOCK4* deletion.

In order to investigate the effect of the *ZNF277* microdeletion and its relationship to a nearby *IMMP2L*_*DOCK4* microdeletion independently described in an autistic family in a previous publication^20^ (Figure 3a), we assessed the effects of *ZNF277* microdeletion on the expression of *ZNF277*, *DOCK4* and *IMMP2L.* Conversely, we also assessed the effect of *IMMP2L*_*DOCK4* microdeletions on the expression of *ZNF277*. In its heterozygous form, the *ZNF277* microdeletion decreased the expression of the entire *ZNF277* transcript (*P*=0.035) but did not significantly alter the expression of *DOCK4* or *IMMP2L* (Figure 3b). Similarly, the microdeletion within *DOCK4* and *IMMP2L* did not decrease the *ZNF277* expression (Figure 3c). Note that this deletion has previously been shown to decrease the expression level of *DOCK4*.^20^

## Discussion

In this study, we identified a novel homozygous microdeletion of exon 5 of the *ZNF277* gene in a child with SLI. Screening of an additional 321 SLI families indicated that the allelic frequency of *ZNF277* microdeletions was more than twice that was observed in control cohorts (1.1 *vs* 0.4%), although the rarity of the microdeletion meant that this difference did not reach significance when examined with a two-tailed Fisher's exact test (*P*=0.206).

Given prior observations of phenotypic and genetic overlaps between SLI and autism, as well as the genomic position of the *ZNF277* gene within a known ASD risk locus, we postulated that the disruption of this gene may be relevant for both disorders. However, screening of a cohort of ASD multiplex families found that the frequency of *ZNF277* microdeletions in individuals with autism was similar to that observed in controls. The *ZNF277* microdeletion that we describe was not documented in the DGV, possibly because of the small number of standard array SNPs contained within the deleted segment. However, we specifically searched Supplementary Data from CNV studies of ASD and found that the microdeletion had been previously characterized during a CNV screen of the Simons Simplex Collection^33^ and occurred at a frequency of 0.3% (6/2248 chromosomes). This figure matches with that observed in our ASD cohort, supporting the current data set and reinforcing our conclusion that the *ZNF277* microdeletion does not contribute to ASD susceptibility.

In the SLI families where DNA of both parents was available, we observed that none of the *ZNF277* microdeletions was *de novo* and that, in many cases, the segregation with language impairment was incomplete. In three families, the microdeletion was not inherited by the proband, whereas in another three families, the microdeletion was inherited by unaffected siblings (Figure 2). Furthermore, although sample sizes of individuals carrying the deletion were small, we did not observe any correlation between the presence of the deletion and any language-related phenotypes investigated (Supplementary Table 1). These data suggest that the heterozygous copy loss of exon 5 of *ZNF277* may represent a risk factor rather than a highly penetrant variant, whereas the homozygous copy loss appears to have a greater impact on the SLI susceptibility. In neurodevelopmental disorders, it is hypothesized that multiple common and rare variants act in concert to determine the phenotype in a complex manner. Under this hypothesis, it is perhaps not surprising to find risk variants in both affected and unaffected family members, or to observe transmission to only a subset of affected individuals of the family. Such findings are consistent with a multigenic threshold model.^34^ Some variants may be highly penetrant while others may be individually insufficient to cause the disorder, but they may combine with other risk loci and/or environmental factors to cross the risk threshold. Even well-established risk loci for autism, ID, schizophrenia and other neurodevelopmental syndromes provide examples of imperfect segregation, as shown by exonic CNVs in *NRXN1*,^35^ missense mutations in *SHANK2*,^36^ rare sequence and structural variants in *CNTNAP2*,^37, 38^ microdeletions and microduplications at 16p11.2.^39, 40, 41^

Given these data, some researchers propose a ‘dual-hit' model.^42^ Under such a hypothesis, the phenotypic effects of copy number events, even those of high penetrance, may be modulated by a second independent genetic ‘insult' which may take the form of an additional CNV or a rare coding mutation. Support for this model comes from studies of single language-impaired cases^43^ and larger cohorts of individuals with ASD^44, 45^ or particular microdeletion/duplication syndromes.^42, 46, 47, 48^ In the current discovery family, there were no obviously co-segregating second hits. A rare duplication of 72 Kb was observed to occur only in the proband (chr2:g.41263841-41336618dup, hg19 – Supplementary Table 2) and a novel 9Kb deletion was observed in the mother and sister (chr2:g.125099924-125109738del, hg19 – Supplementary Table 2). However neither of these events affects any coding sequence. Larger or more in-depth studies would therefore be required to further investigate possible genetic modulators of *ZNF277* microdeletions. It is conceivable that the phenotypic variability associated with heterozygous *ZNF277* microdeletions might be modulated by a combination of pathogenic single-nucleotide variants in other genomic locations, small changes in regulatory regions or other factors beyond the scope of this study. We found that the heterozygous microdeletion of exon 5 in *ZNF277* does not affect the expression of autism candidate genes *IMMP2L* and *DOCK4*. Similarly, a microdeletion involving the 3′ end of *DOCK4* (exons 27–52), a gene which lies head-to-head with the *ZNF277* gene, and the first three exons of *IMMP2L*, decreases the expression level of *DOCK4* but not *ZNF277*. Taken together, these data suggest that *ZNF277* microdeletions may have a role in SLI susceptibility that is distinct from the autism risk loci described in the *AUTS1* region.

*ZNF277* is an evolutionary conserved zinc finger gene with 12 exons.^49^ It is expressed in several tissues, including the brain, particularly in the neocortex and hippocampus in early mid-fetal development.^50^ Although the function of *ZNF277* has not been studied in humans, the mouse *Zfp277* gene, which shows >80% homology to the human gene at the amino-acid level, has been implicated in the epigenetic regulation of cellular memory.^51^ *Zfp277^−/−^* mice were born healthy and fertile, indicating that the knockout is not lethal,^51^ consistent with our finding of viability for humans with no functional *ZNF277*. Interestingly, the Zfp277 protein directly interacts with Bmi-1, a key component of the Polycomb Repressor Complex. This complex has an important role in the maintenance of adult stem cells from numerous tissues, including the central nervous system.^52, 53^

Sequencing of the breakpoints in the discovery individual demonstrated that the breakpoint boundaries lie in two LINE elements, L2c and L1M4. L2c (chr7:g.111941666_111941883, hg19, strand +) belongs to the L2 LINE family and L1M4 (chr7:g.111961275_111963848, hg19, strand −) to the L1 LINE family, which promote structural variation through non-allelic homologous recombination. BLAST alignment of the entire sequence of these two elements did not reveal extended homology between them. However, sequencing of the breakpoints revealed 2 bp microhomology at the junctions, suggesting that this deletion may to be generated through a microhomology-mediated repair mechanism.^54^ In conclusion, we propose that the disruption of *ZNF277* may contribute to SLI in a complex genetic model. We further hypothesize that this risk is distinct from the autism risk loci as previously described in this region. Further studies will be required to replicate these findings and characterize the function of the human protein ZNF277, clarifying its potential implication in language development.

## NOTE ADDED IN PROOF

During the proof-editing stage of the manuscript, we noted that a deletion of the same size had also been reported in the most recent release of DGV (evs2656841) (DGV, January 2014). These data again supported the expected frequencies found throughout our study (evs2656841 frequency=0.35% – 8 losses in 2302 chromosomes (1151 individuals)).

## Data Archiving

The CNV data generated for the discovery family will be archived in the DGVa (http://www.ebi.ac.uk/dgva/).

## Notes

The WGS500 Consortium is partly funded by Illumina. DR Bentley is an employee of Illumina Inc.
