# Supplementary material for: Homozygous microdeletion of exon 5 in ZNF277 in a girl with specific language impairment
Source: Eur J Hum Genet. 2014 Feb 12;22(10):1165–71. doi: 10.1038/ejhg.2014.4 (PMC4169542; doi:10.1038/ejhg.2014.4)
Supplement: Supplementary Table 1 [file ejhg20144x1.pdf]

Supplementary Table 1
Quantitative statistics for individuals carrying ZNF277 deletions

	Total no. individuals	no. individuals carrying the deletion	no. individuals without the deletion	allelic frequency of the deletion
**samples**	1233	18	1215	0.007
**parents**	545	8	537	0.007
**probands**	317	5	312	0.008
**sibs**	371	5	366	0.007
**families**	322	9	313	0.014

	ELS	RLS	NWR	READ	SPELL	PIQ	VIQ
**no. phenotyped individuals carrying deletion**	**7**	**7**	**15**	**3**	**3**	**8**	**6**
**no. phenotyped individuals without deletion**	**573**	**575**	**835**	**274**	**272**	**612**	**478**
**mean phenotype in individuals carrying deletion**	82.00	93.29	87.93	101.00	99.33	94.63	93.17
**mean phenotype in individuals without deletion**	78.49	88.33	90.43	92.86	91.79	100.13	95.59
**t-test P (2 tail, equal variance)**	0.58	0.46	0.61	0.36	0.41	0.32	0.73

ELS - CELF expressive composite score
RLS - CELF recpetive composite score
NWR - nonword repetition
READ - Wechsler Objective Reading Dimensions (WORD) single word reading
SPELL - Wechsler Objective Reading Dimensions (WORD) single word spelling
PIQ - Wechsler Intelligence Scales for Children (WISC) performance (non-verbal) IQ
VIQ - Wechsler Intelligence Scales for Children (WISC) verbal IQ
NOTE - Only the NWR data were available for parents, therefore the test data shown is based primarily on children carrying the deletion
NOTE - these calculations exclude the discovery individual with the homozygous ZNF277 deletion
