# Supplementary material for: Homozygous microdeletion of exon 5 in ZNF277 in a girl with specific language impairment
Source: Eur J Hum Genet. 2014 Feb 12;22(10):1165–71. doi: 10.1038/ejhg.2014.4 (PMC4169542; doi:10.1038/ejhg.2014.4)
Supplement: Supplementary Table 2 [file ejhg20144x2.pdf]

**Supplementary Table 2**
**All CNVs called in members of the discovery family**

| Individual in Discovery Pedigree | Chr | Start (hg19) | End (hg19) | No. SNPs | Size | Deletion/ Duplication | Start SNP | End SNP | Confidence | Genes overlapped | Coding? | No. overlapping events in DGV (January 2012) | Classification |
|---|---|---|---|---|---|---|---|---|---|---|---|---|---|
| G4_2 | chr2 | 34,699,812 | 34,726,904 | 8 | 27093 | deletion | rs7594384 | rs13392627 | 30.935 | Intergenic | | >5 events or >50% overlap with any event | common |
| G4_4 | chr2 | 41,238,321 | 41,248,468 | 6 | 10148 | deletion | rs7571620 | rs7589532 | 10.8313 | Intergenic | | >5 events or >50% overlap with any event | common |
| G4_4 | chr2 | 41,263,841 | 41,336,618 | 15 | 72778 | duplication | rs12989241 | rs2374143 | 55.216 | Intergenic | | 2, both less than 50% overlap | rare |
| G4_2 | chr2 | 125,099,924 | 125,109,738 | 6 | 9815 | deletion | rs12475383 | rs7560392 | 17.8278 | CNTNAP5 | No | none | novel |
| G4_5 | chr2 | 125,099,924 | 125,109,738 | 6 | 9815 | deletion | rs12475383 | rs7560392 | 18.2617 | CNTNAP5 | No | none | novel |
| G4_5 | chr2 | 213,187,034 | 213,191,389 | 8 | 4356 | deletion | rs10164675 | rs1505366 | 25.295 | ERBB4 | No | >5 events or >50% overlap with any event | common |
| G4_4 | chr2 | 213,187,034 | 213,191,389 | 8 | 4356 | deletion | rs10164675 | rs1505366 | 26.58 | ERBB4 | No | >5 events or >50% overlap with any event | common |
| G4_4 | chr3 | 162,130,691 | 162,142,475 | 7 | 11785 | deletion | rs4856685 | rs6803545 | 21.6863 | Intergenic | | >5 events or >50% overlap with any event | common |
| G4_2 | chr4 | 138,093,809 | 138,097,449 | 3 | 3641 | deletion | rs7691001 | rs11929727 | 14.106 | Intergenic | | >5 events or >50% overlap with any event | common |
| G4_5 | chr4 | 161,881,000 | 161,884,036 | 3 | 3037 | deletion | rs7662611 | rs13120999 | 12.46 | Intergenic | | >5 events or >50% overlap with any event | common |
| G4_5 | chr5 | 7,178,644 | 7,191,074 | 7 | 12431 | deletion | rs11745983 | rs4702440 | 11.1036 | Intergenic | | >5 events or >50% overlap with any event | common |
| G4_3 | chr5 | 9,902,403 | 9,924,597 | 16 | 22195 | deletion | rs7732991 | rs11949879 | 30.2506 | LOC285692 | Yes | >5 events or >50% overlap with any event | common |
| G4_1 | chr5 | 9,902,403 | 9,924,597 | 16 | 22195 | deletion | rs7732991 | rs11949879 | 39.631 | LOC285692 | Yes | >5 events or >50% overlap with any event | common |
| G4_5 | chr5 | 9,902,403 | 9,924,597 | 16 | 22195 | deletion | rs7732991 | rs11949879 | 41.9547 | LOC285692 | Yes | >5 events or >50% overlap with any event | common |
| G4_4 | chr5 | 155,477,866 | 155,488,438 | 5 | 10573 | deletion | rs998997 | rs10070956 | 11.9644 | Intergenic | | >5 events or >50% overlap with any event | common |
| G4_1 | chr7 | 44,935 | 68,920 | 13 | 23986 | duplication | rs7456436 | rs10272797 | 13.0095 | Intergenic | | >5 events or >50% overlap with any event | common |
| G4_4 | chr7 | 111,955,948 | 111,960,100 | 3 | 4153 | deletion | rs11769219 | rs7802828 | 13.585 | ZNF277 | Yes | 1 large event with 100% overlap | novel |
| G4_1 | chr7 | 111,955,948 | 111,960,100 | 3 | 4153 | deletion | rs11769219 | rs7802828 | not predicted by algorithms but identified by q-PCR | ZNF277 | Yes | 1 large event with 100% overlap | novel |
| G4_2 | chr7 | 111,955,948 | 111,960,100 | 3 | 4153 | deletion | rs11769219 | rs7802828 | 12.171 | ZNF277 | Yes | 1 large event with 100% overlap | novel |
| G4_2 | chr8 | 51,031,221 | 51,033,517 | 4 | 2297 | deletion | rs17687439 | rs203617 | 17.978 | SNTG1 | No | >5 events or >50% overlap with any event | common |
| G4_4 | chr9 | 8,009,428 | 8,014,674 | 8 | 5247 | deletion | rs9408721 | rs9299059 | 14.1357 | Intergenic | | >5 events or >50% overlap with any event | common |
| G4_2 | chr9 | 8,009,428 | 8,014,674 | 8 | 5247 | deletion | rs9408721 | rs9299059 | 15.3321 | Intergenic | | >5 events or >50% overlap with any event | common |
| G4_5 | chr9 | 8,009,428 | 8,014,674 | 8 | 5247 | deletion | rs9408721 | rs9299059 | 19.6495 | Intergenic | | >5 events or >50% overlap with any event | common |
| G4_3 | chr9 | 8,009,428 | 8,014,674 | 8 | 5247 | deletion | rs9408721 | rs9299059 | 20.3505 | Intergenic | | >5 events or >50% overlap with any event | common |
| G4_4 | chr10 | 42,794,125 | 42,810,976 | 3 | 16852 | deletion | rs10899976 | rs12412635 | 12.0003 | Intergenic | | >5 events or >50% overlap with any event | common |
| G4_5 | chr12 | 63,946,056 | 64,088,345 | 14 | 142290 | duplication | rs3884364 | rs4107159 | 33.7772 | DPY19L2 | Yes | >5 events or >50% overlap with any event | common |
| G4_2 | chr12 | 63,979,105 | 64,101,324 | 12 | 122220 | duplication | rs7316001 | rs11175134 | 33.7005 | DPY19L2 | Yes | >5 events or >50% overlap with any event | common |
| G4_3 | chr13 | 32,533,830 | 32,535,996 | 4 | 2167 | deletion | rs8002033 | rs449421 | 15.103 | Intergenic | | >5 events or >50% overlap with any event | common |
| G4_4 | chr13 | 32,533,830 | 32,535,996 | 4 | 2167 | deletion | rs8002033 | rs449421 | 17.037 | Intergenic | | >5 events or >50% overlap with any event | common |
| G4_4 | chr13 | 70,735,399 | 70,773,398 | 17 | 38000 | deletion | rs2498516 | rs1999074 | 63.409 | Intergenic | | >5 events or >50% overlap with any event | common |
| G4_1 | chr13 | 70,744,882 | 70,773,398 | 16 | 28517 | deletion | rs1999078 | rs1999074 | 66.51 | Intergenic | | >5 events or >50% overlap with any event | common |
| G4_3 | chr15 | 34,718,594 | 34,807,851 | 12 | 89258 | deletion | rs3894644 | rs7176678 | 12.7059 | GOLGA8A | Yes | >5 events or >50% overlap with any event | common |
| G4_2 | chr15 | 34,730,758 | 34,807,851 | 11 | 77094 | deletion | rs12439426 | rs7176678 | 23.7937 | Intergenic | | >5 events or >50% overlap with any event | common |
| G4_5 | chr17 | 34,458,934 | 34,468,886 | 12 | 9953 | duplication | rs2158114 | rs12942764 | 12.8744 | Intergenic | | >5 events or >50% overlap with any event | common |
| G4_4 | chr17 | 34,458,934 | 34,476,396 | 15 | 17463 | duplication | rs2158114 | rs16972085 | 31.256 | Intergenic | | >5 events or >50% overlap with any event | common |
| G4_2 | chr18 | 63,729,570 | 63,730,950 | 3 | 1381 | deletion | rs17276824 | rs2541790 | 13.821 | Intergenic | | >5 events or >50% overlap with any event | common |
| G4_1 | chr22 | 25,686,040 | 25,928,629 | 55 | 242590 | duplication | rs6004549 | rs6004694 | 65.1091 | CRYBB2P1,IGLL3P,LRP5L | Yes | >5 events or >50% overlap with any event | common |
| G4_4 | chr22 | 25,753,777 | 25,905,668 | 27 | 151892 | duplication | rs5996921 | rs2013369 | 32.757 | CRYBB2P1,LRP5L | Yes | >5 events or >50% overlap with any event | common |
| G4_3 | chr22 | 25,756,674 | 25,909,255 | 26 | 152582 | duplication | rs133198 | rs16980329 | 73.1422 | CRYBB2P1,LRP5L | Yes | >5 events or >50% overlap with any event | common |
| G4_3 | chrX | 116,083,902 | 116,091,292 | 5 | 7391 | duplication | rs5910598 | rs4524978 | 13.473 | Intergenic | | >5 events or >50% overlap with any event | common |

This list includes all CNVs called in all individuals of the discovery family by both Penn CNV and QuantiSNP with a Bayes Factor confidence of >10.

CNVs are classified as novel if they do not overlap >50% with any events in the DGV (january 2012), rare if they do not overlap >50% with >5 events in the DGV (January 2012) and common if they overlap >50% with any event or <50% with >5 events in the DGV (January 2012).
