# Supplementary material for: Homozygous microdeletion of exon 5 in ZNF277 in a girl with specific language impairment
Source: Eur J Hum Genet. 2014 Feb 12;22(10):1165–71. doi: 10.1038/ejhg.2014.4 (PMC4169542; doi:10.1038/ejhg.2014.4)
Supplement: Supplementary Information [file ejhg20144x3.pdf]

SLI Consortium (SLIC) Members:

DF Newbury, R Nudel, AP Monaco, NH Simpson; The Wellcome Trust Centre for Human Genetics, Roosevelt Drive, Headington, Oxford
G Baird, V Slonims; Newcomen Centre, Evelina Children's Hospital, London
A Clark, J Watson; Speech and Hearing Sciences, Queen Margaret University, Edinburgh
A O'Hare; Child Life and Health, University of Edinburgh, Edinburgh
G Conti-Ramsden, Z Simkin; School of Psychological Sciences, University of Manchester
P Bolton, E Simonoff, A Pickles; Institute of Psychiatry, London
D.V.M. Bishop; Department of Experimental Psychology, University of Oxford
SE Fisher; Max Planck Institute for Psycholinguistics, Nijmegen, Netherlands
A Everitt, E.R. Hennessy; University Child Health and DMDE, University of Aberdeen
J Seckl; Molecular Medicine Centre, University of Edinburgh
H Cowie; Department of Speech and Language Therapy, Royal Hospital for Sick Children, Edinburgh
W Cohen; Psychological Sciences and Health, University of Strathclyde
J Nasir; Division of Biomedical Sciences, St George's University of London

IMGSAC Members

UK

Jeremy R Parr; Institute of Neuroscience, Newcastle University, UK
A S Le-Couteur; Institute of Health and Society, Newcastle University, UK
Gillian Baird; Guy's and St Thomas' NHS Trust & King's College London, UK
Andrew Pickles, Patrick Bolton; Institute of Psychiatry, King's College London, UK
Alistair Pagnamenta, Richard Holt, Inês Girão Meireles de Sousa, Nuala Simpson; Wellcome Trust Centre for Human Genetics, University of Oxford, UK
Simon Wallace; University of Oxford Department of Psychiatry, UK
Kerstin Wittemeyer; School of Education, University of Birmingham, UK
Jonathan Green; Academic Department of Child Psychiatry, University of Manchester, Manchester, UK
Janine Lamb; Centre for Integrated Genomic Medical Research, The University of Manchester, Manchester, UK

EU

Sabine Klauck; Division of Molecular Genome Analysis, German Cancer Research Center (DKFZ), Heidelberg, Germany
Fritz Poutska; Department of Child and Adolescent Psychiatry, Psychosomatics and Psychotherapy, J.W. Goethe University Frankfurt, Germany
Maretha de Jonge, Herman van Engeland; Department of Child Psychiatry, Utrecht, The Netherlands
Bernadette Roge; Centre d'Etudes et de Recherches en Psychopathologie, Toulouse, France
Sven Bolte; Department of Women's and Children's Health, Karolinska Institutet, Stockholm, Sweden
Lennart Pedersen; Center for Autisme, Denmark
Torban Isager; University Centre for Child and Adolescent Psychiatry Vibeholmsvej, Glostrup, Denmark
Elena Maestrini, Elena Baccelli; Dipartimento di Farmacia e BioTecnologie
Università di Bologna
John Tsiantis; University Department of Child Psychiatry, Athens, Greece

Canada

Anthony Bailey; Department of Psychiatry, University of British Columbia, Vancouver, Canada

USA

Anthony Monaco; Tufts University, Boston, Massachusetts, USA
Edwin Cook ; Institute for Juvenile Research, Department of Psychiatry, University of Illinois at Chicago, Chicago, USA

Steven Guter; Department of Psychiatry, University of Illinois, Chicago, Illinois, USA
Fred Volkmar; Child Study Centre, Yale University, New Haven, Connecticut, USA
Catherine Lord; Weill Cornell Medical College/ New York Presbyterian Hospital, Columbia University, White Plains, NY, USA
Bennett Leventhal; Nathan Kline Institute for Psychiatric Research (NKI), Orangeberg, NY, USA
Eric Fombonne; Oregon Health & Science University, Institute for Development & Disability, Department of Psychiatry, Portland, USA

WGS500 AUTHORSHIP (05.07.2012)

Steering Committee

Peter Donnelly (Chair)[1], John Bell[2] , David Bentley[3], Gil McVean[1], Peter Ratcliffe[1], Jenny Taylor[1,4], Andrew Wilkie[4, 5]

Operations Committee

Peter Donnelly[1] (Chair) John Broxholme[1], David Buck[1], Jean-Baptiste Cazier[1], Richard Cornall[1], , Lorna Gregory[1], Julian Knight[1], Gerton Lunter[1], Gilean McVean[1], Jenny Taylor[1,4], Ian Tomlinson[1, 4], Andrew Wilkie[4, 5]

Sequencing & Experimental Follow up

David Buck[1] (Lead) Christopher Allan[1], Moustafa Attar[1], Angie Green[1], Lorna Gregory[1], Sean Humphray[3,] Zoya Kingsbury[3], Sarah Lamble[1], Lorne Lonie[1], Alistair Pagnamenta[1], Paolo Piazza[1], Guadelupe Polanco[1], Amy Trebes[1]

Data Analysis

Gil McVean[1] (Lead), Peter Donnelly[1], Jean-Baptiste Cazier[1], John Broxholme[1], Richard Copley[1], Simon Fiddy[1], Russell Grocock[3], Edouard Hatton[1], Chris Holmes[1], Linda Hughes[1], Peter Humburg[1], Alexander Kanapin[1], Stefano Lise[1], Gerton Lunter[1], Hilary Martin[1], Lisa Murray[3] , Davis McCarthy[1], Andy Rimmer[1], Natasha Sahgal[1], Ben Wright[1], Chris Yau[6]

[1] The Wellcome Trust Centre for Human Genetics, Roosevelt Drive, Oxford, OX3 7BN, UK.
[2] Office of the Regius Professor of Medicine, Richard Doll Building, Roosevelt Drive, Oxford, OX3 7LF, UK
[3] Illumina Cambridge Ltd., Chesterford Research Park, Little Chesterford, Essex, CB10 1XL, UK
[4] NIHR Oxford Biomedical Research Centre, Oxford, UK.
[5] Weatherall Inst of Molecular Medicine, University of Oxford; John Radcliffe Hospital Headington, Oxford OX3 9DS, UK
[6] Imperial College London, South Kensington Campus, London, SW7 2AZ. UK
